# Supplementary material for: Platelet Counts and Risk of Severe Retinopathy of Prematurity: A Bayesian Model-Averaged Meta-Analysis
Source: Children (Basel). 2023 Dec 8;10(12):1903. doi: 10.3390/children10121903 (PMC10741847; doi:10.3390/children10121903)
Supplement: Supplementary file 1 [file children-10-01903-s001.zip › children-2734270-supplementary.pdf]

Supplementary material

Platelet Counts and Risk of Severe Retinopathy of Prematurity: A Bayesian Model-Averaged Meta-analysis

Mohamad F. Almutairi[1], Silvia Gulden[2], Tamara M. Hundscheid[1], František Bartoš[3], Giacomo Cavallaro[4], and Eduardo Villamor[1*]

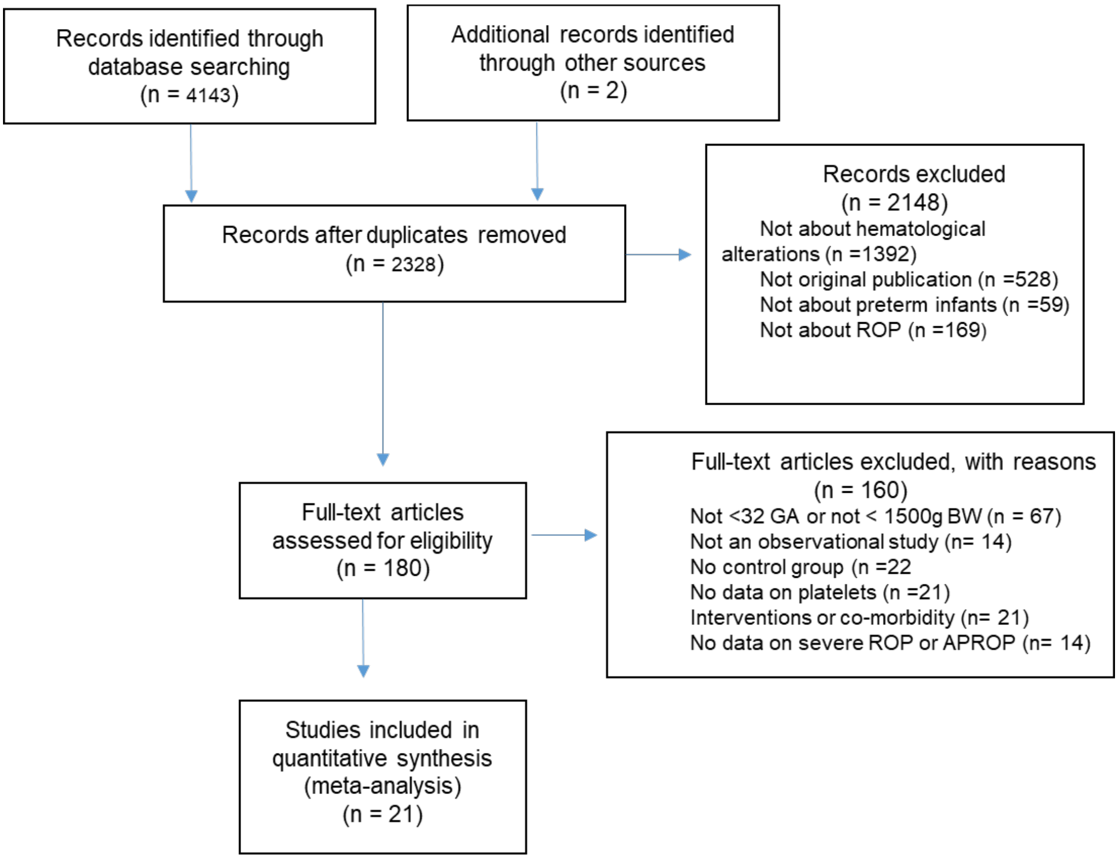

Figure S1. Search flow diagram

Table S1. Search strategy

**PubMed**	
#1	(("Retinopathy of Prematurit*"[tiab]) OR (ROP[tiab]) OR ("Prematurity Retinopathy" [tiab]) OR ("Retrolental Fibroplasia*" [tiab]) OR ("Retinopathy of Prematurity"[Mesh]) OR ("Terry syndrome"[tiab]))
#2	(("premature neonate"[tiab] OR "preterm neonate"[tiab] OR "premature infant*" [tiab]) OR ("preterm infant*"[tiab]) OR ("extremely premature infant*"[tiab]) OR ("extremely preterm infant*"[tiab]) OR ("Infant, Premature"[Mesh]) OR ("low birth weight infant*"[tiab]) OR("low birth weight*"[tiab]) OR("very low birth weight infant*"[tiab]) OR ("very low birth weight*"[tiab]) OR ("Extremely Low Birth Weight infant*"[tiab]) OR ("Extremely Low Birth Weight*"[tiab]) OR ("Infant, Low Birth Weight"[Mesh]) OR ("Decreased gestational age" [tiab]) OR ("premature gestation" [tiab]) OR ("prematurity" [tiab]) OR ("preterm"[tiab]) OR ("premature"[tiab])))
#3	(("risk factor*"[tiab]) OR ("platelet count"[MeSH]) OR ("Platelet Count"[tiab]) OR ("platelet transfusion"[MeSH]) OR ("mean platelet volume"[MeSH]) OR ("Mean Platelet Volume"[tiab]) OR ("Platelet Transfusion"[tiab]) OR ("thrombocytopenia"[MeSH]) OR ("Thrombocytopenia"[tiab]))
#4	#1 AND #2 AND #3
**EMBASE**	
#1	exp retinopathy of prematurity/ or (retinopathy of prematurity or retrolental dysplasia or retrolental fibrosis or retrolenticular fibroplasia).ab,ti,kf.
#2	exp prematurity/ or extremely premature infant*.ab,kf,ti. or infant*, extremely premature.ab,kf,ti. or (preterm* OR pre-term* OR prematur* OR pre-matur*).ti,ab,kf. or infant*, premature.ab,kf,ti. or infant*, premature, disease*.ab,kf,ti. or neonate*, premature.ab,kf,ti. or pre-mature infant*.ab,kf,ti. or pre-term baby*.ab,kf,ti. or pre-term child*.ab,kf,ti. or pre-term infant*.ab,kf,ti. or pre-term neonate*.ab,kf,ti. or pre-term newborn*.ab,kf,ti. or premature.ab,kf,ti. or premature baby*.ab,kf,ti. or premature birth*.ab,kf,ti. or premature child*.ab,kf,ti. or premature childbirth*.ab,kf,ti. or premature infant*.ab,kf,ti. or premature infant* disease*.ab,kf,ti. or premature

	neonate*.ab,kf,ti. or premature newborn*.ab,kf,ti. or premature syndrome*.ab,kf,ti. or prematuritas.ab,kf,ti. or preterm baby*.ab,kf,ti. or preterm child*.ab,kf,ti. or preterm infant*.ab,kf,ti. or preterm neonate*.ab,kf,ti. or preterm newborn*.ab,kf,ti. or exp anemia of prematurity/ or anaemia of prematurity.ab,kf,ti. or premature anaemia.ab,kf,ti. or premature anemia.ab,kf,ti. or prematurity anaemia.ab,kf,ti. or prematurity anemia.ab,kf,ti. or newborn anemia.ab,kf,ti.
#3	exp risk factor/ or risk factor*.ab,kf,ti. or exp blood transfusion/ or exp erythrocyte transfusion/ or normocyte transfusion.ab,kf,ti. or red blood cell transfusion.ab,kf,ti. or transfusion*, erythrocyte*.ab,kf,ti. or exp erythrocyte count/ or counting, erythrocyte.ab,kf,ti. or erythrocyte counting.ab,kf,ti. or erythrocyte number*.ab,kf,ti. or normocyte count.ab,kf,ti. or normocyte number.ab,kf,ti. or RBC count.ab,kf,ti. or red blood cell count.ab,kf,ti. or red blood cell number*.ab,kf,ti. or red cell count.ab,kf,ti. or red cell number*.ab,kf,ti. or exp iron deficiency anemia/ or anaemia, iron deficiency.ab,kf,ti. or anaemia, microcytic hypochromic.ab,kf,ti. or iron deficient anaemia.ab,kf,ti. or iron deficiency anaemia.ab,kf,ti. or exp platelet count/ or blood platelet count.ab,kf,ti. or count, blood platelet*.ab,kf,ti. or count, thrombocytic.ab,kf,ti. or platelet counting.ab,kf,ti. or platelet number*.ab,kf,ti. or exp thrombocytopenia/ or thrombocyte* count.ab,kf,ti. or thrombocyte counting.ab,kf,ti. or thrombocyte number*.ab,kf,ti. or thrombocytic count.ab,kf,ti. or thrombocyte/ or blood platelet.ab,kf,ti. or blood platelet*.ab,kf,ti. or platelet*.ab,kf,ti. or exp mean platelet volume/ or average platelet volume.ab,kf,ti. or average thrombocyte volume.ab,kf,ti. or mean thrombocyte volume.ab,kf,ti. or thrombocyte transfusion/ or blood platelet transfusion*.ab,kf,ti. or platelet concentrate transfusion*.ab,kf,ti. or platelet transfusion*.ab,kf,ti. or thrombocytic transfusion.ab,kf,ti. or transfusion, thrombocyte*.ab,kf,ti.
#4	#1 AND #2 AND #3
**Web of Science**	
#2	(((TI="premature neonate" OR AB="premature neonate") OR (TI="preterm neonate" OR AB="preterm neonate") OR (TI="premature infant*" OR AB="premature infant*")) OR ((TI="preterm infant*" OR AB="preterm infant*")) OR ((TI="extremely premature infant*" OR AB="extremely premature infant*")) OR ((TI="extremely preterm infant*" OR

	AB="extremely preterm infant*")) OR (ALL="Infant, Premature") OR ((TI="low birth weight infant*" OR AB="low birth weight infant*")) OR ((TI="low birth weight*" OR AB="low birth weight*")) OR ((TI="very low birth weight infant*" OR AB="very low birth weight infant*")) OR ((TI="very low birth weight*" OR AB="very low birth weight*")) OR ((TI="Extremely Low Birth Weight infant*" OR AB="Extremely Low Birth Weight infant*")) OR ((TI="Extremely Low Birth Weight*" OR AB="Extremely Low Birth Weight*")) OR (ALL="Infant, Low Birth Weight") OR ((TI="Decreased gestational age" OR AB="Decreased gestational age")) OR ((TI="premature gestation" OR AB="premature gestation")) OR ((TI=prematurity OR AB=prematurity)) OR ((TI=preterm OR AB=preterm)) OR ((TI=premature OR AB=premature)))
#3	(((TI="risk factor*" OR AB="risk factor*")) OR (ALL="Blood Transfusion") OR ((TI="blood transfusion*" OR AB="blood transfusion*")) OR ((TI="blood component transfusion*" OR AB="blood component transfusion*")) OR ((TI="Erythrocyte Transfusion*" OR AB="Erythrocyte Transfusion*")) OR ((TI="red blood cell transfusion*" OR AB="red blood cell transfusion*")) OR ((TI="red cell transfusion*" OR AB="red cell transfusion*")) OR ((TI="red blood cell count" OR AB="red blood cell count")) OR ((TI="RBC count" OR AB="RBC count")) OR (ALL="anemia, iron-deficiency") OR (ALL=iron) OR ((TI="iron deficienc*" OR AB="iron deficienc*")) OR ((TI=anemia OR AB=anemia)) OR (ALL="platelet count") OR ((TI="Platelet Count" OR AB="Platelet Count")) OR (ALL="platelet transfusion") OR (ALL="mean platelet volume") OR ((TI="Mean Platelet Volume" OR AB="Mean Platelet Volume")) OR ((TI="Platelet Transfusion" OR AB="Platelet Transfusion")) OR (ALL=thrombocytopenia) OR ((TI=Thrombocytopenia OR AB=Thrombocytopenia)))
#4	#1 AND #2 AND #3

Table S2. Characteristics of the included studies and risk of bias**.**

First author, year	Country	Design	Prospective ?	Centers	n	Mean or median GA	NOS score
Abdel Salaam Gomaa, 2022	Egypt	Ca-Co	No	1	50	31.2	7
Akyuz Unsal, 2020	Turkey	Ca-Co	No	1	36	28,9	8
Cakir, 2018	Sweden	Cohort	No	2	202	<27	7
Cekmez, 2013	Turkey	Cohort	Yes	1	171	30.5	
Celik, 2021	Turkey	Ca-Co	No	1	46	26.8	7
Choreziak, 2022	Poland	Cohort	No	1	163	26.6	7
Fevereiro-Martins, 2023	Portugal	Cohort	Yes	8	455	29.6	8
Hellgren, 2021	Sweden	Cohort	No	1	78	25.2	7
Hengartner, 2020	Switzerland	Ca-Co	No	9	356	25,5	8
Jensen, 2011	USA	Ca-Co	No	1	182	25.6	8
Korkmaz, 2018	Turkey	Ca-Co	No	1	146	27.5	8
Lim, 2021	Malysia	Ca-Co	No	1	93	27.6	8
Lundgren, 2017	Sweden	Ca-Co	No	1	18	24.4	8
Okur, 2016	Turkey	Cohort	No	1	330	28	7
Panchal, 2020	Australia	Ca-Co	No	1	227	25.1	?
Parrozzani, 2021	Italy	Cohort	No	1	529	29.0	8
Sahinoglu-Keskek, 2020	Turkey	Cohort	No	1	137	30.2	7
Sancak, 2019	Turkey	Ca-Co	No	1	162	27.6	8
Tao, 2015	China	Ca-Co	No	1	148	29.9	7
Vinekar, 2010	India	Ca-Co	No	1	31	29.4	8
Yuksul 2014	Turkey	Ca-Co	No	1	65	28.9	7
